# Full Complex-Amplitude Modulation of Surface Waves Based on Spin-Decoupled Metasurface

**DOI:** 10.3390/mi14081511

**Published:** 2023-07-27

**Authors:** Quan Li, Chao Wu, Yu Xie, Song Li, Hongqiang Li, Lijun Jin

**Affiliations:** 1 College of Electronic and Information Engineering, Tongji University, Shanghai 200092, China; 1210583@tongji.edu.cn (Q.L.); hqlee@tongji.edu.cn (H.L.); jinlj@tongji.edu.cn (L.J.); 2 The Institute of Dongguan—Tongji University, Dongguan 523808, China; 3 China North Industries Corporation, Beijing 100053, China; xieyu1219@163.com; 4 State Key Laboratory of Advanced Fiber Composite, Beijing 102101, China; lisong1996@126.com

**Keywords:** surface wave, metasurface, spin-decouple

## Abstract

This work proposes a method for surface wave (SW) coupling along with flexible complex amplitude modulation of its wavefront. The linearly polarized incident plane wave is coupled into the surface mode with complex wavefront by exploiting the spin-decouple nature of a reflective chiral meta-atom. As verification, two kinds of metasurface couplers are designed. The first kind contains two examples for SW airy beam generation with and without deflection under linearly polarized illumination, respectively. The second kind is a bi-functional device capable of SW focusing under left-handed circularly polarized illumination, and propagating wave deflection under right-handed circularly polarized illumination, respectively, to verify the fundamental spin-decoupled character. Simulated and experimental results are in good agreement. We believe that this method provides a flexible approach for complex SW applications in integrated optics, optical sensing, and other related fields.

## 1. Introduction

As a special bounded eigenmode at the metal/dielectric interface, surface wave (SW) [1] has been attracting a lot of attention due to its potential in the frontiers of photonics [2,3,4,5,6]. The coupling of the propagating wave (PW) to the SW with a tailored wavefront is crucial for various applications, in which the independent control of the amplitude and phase of the generated SW remains challenging.

Metasurface [7] is one of the most popular platforms for SW coupling and modulation, benefiting from its excellent amplitude and phase control ability on a subwavelength scale. The phase-gradient-metasurface (PGM) [8] has been proposed to realize the PW-to-SW conversion with high efficiency by constructing an artificial transverse phase gradient [8,9,10]. To further introduce the abrupt phase or amplitude shift to the generated SW, other approaches have been explored by altering the initial phase of the PGM [11,12] or utilizing the polarization-dependent coupling efficiency of the inclusions [13,14,15,16,17], respectively. Recently, a novel approach is proposed to realize the arbitrary complex wavefront manipulation of the SW utilizing a stand-along transmissive meta-coupler [18], which can independently control the amplitude and phase of the transmission field of a particular polarization. However, the separate design makes it relatively sensitive to dimensional deviations and is relatively high-profile. A compact meta-device that is capable of independent control of the amplitude and phase of SW is still in great need in complicated and integrated application scenarios.

In this work, a meta-device design method based on reflective metallic Fermat Spirals is proposed to address the aforementioned issue. Based on the theory of Aharonov-Anandan (AA) geometric phase and the Pancharatnam-Berry (PB) [19,20,21] geometric phase, the monolithic meta-device demonstrates the high-efficient SW coupling and phase modulation capability under left-handed circularly polarized (LCP) and right-handed circularly polarized (RCP) illumination by simply altering the orientation angle of the starting point and the overall structure of each spiral, respectively. Furthermore, independent control of the amplitude and phase of the coupled SW can also be realized under linear polarization illumination, which benefits from the spin-decouple nature of the inclusions. Concerning the existing methods, our approach enables the simultaneous coupling and arbitrary complex amplitude controlling of the SW within a monolithic compact meta-device. On the other hand, benefiting from the nature of the AA phase and PB phase, only two orientation angles are needed for the design process, which avoids the time-consuming multi-dimensional parameter searching process. Two kinds of metasurface couplers are numerically and experimentally characterized for verification. The first device of the first kind is for normal SW airy beam generation with finite energy under linear polarization illumination, the second device of the first kind is capable of SW finite-energy airy beam generation with beam deflection of 10° under linear polarization illumination, and the second kind of metasurface coupler is designed for bi-function SW and PW manipulation under the respective illumination of distinct spins. The outcomes provide a new approach for the high-performance SW photonics devices design in different frequency regimes ranging from microwave to optical frequency.

## 2. Concept and Meta-Atom Design

The PGM is an important approach for PW-to-SW conversion by constructing the proper momentum matching [8]. Based on PGM, phase control of the SW wavefront can be further achieved by altering the initial phase of the supercells [11,12]. Following this approach, a polarization-delinked bi-functional meta-device is proposed utilizing the polarization decouple meta-atoms [11]. To achieve the amplitude modulation of the SW, dimensions of the nano-slits including the size and orientation angle are exploited to achieve a continuous amplitude response for specific reflectance components [22].

**Figure 1:**
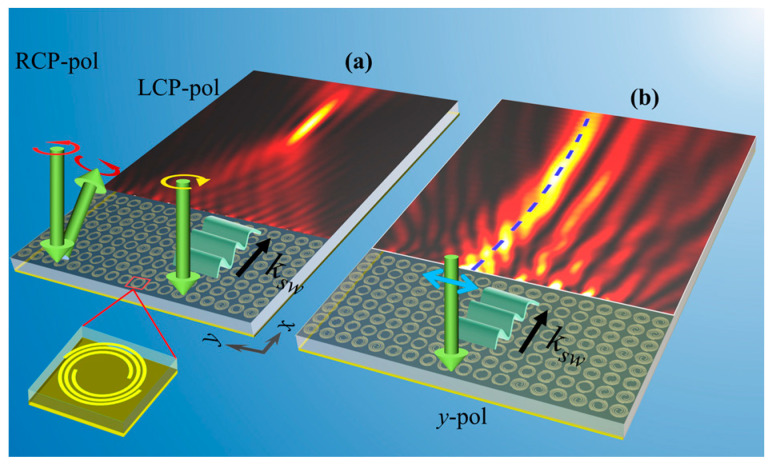
Schematic of complex-amplitude modulation of surface waves. (**a**) Demonstration of the spin-decouple phase control nature of the Fermat spiral structure. Distinct behaviors for different polarization states are encoded into the same device, in which the RCP-polarized incident wave is deflected back to the space with a specially designed angle, while the LCP-polarized incident wave is coupled to the surface mode of the dielectric waveguide. (**b**) Advanced complex amplitude modulation ability of the coupled surface wave that is used to generate the finite-energy airy beam with a main lobe deflection angle of 10°. The blue dashed line indicates the main lobe trace of the airy beam.

In this work, we further realize the full-range independent amplitude and phase control over the SW under linear polarized illumination based on a spin-decoupled meta-atom that follows the AA phase mechanism. The concept of this work is shown in Figure 1. Under linear polarized illumination, the full range amplitude covering [0, 1] and full range phase covering [0, 2*π*) of the coupled SW wavefront can be achieved by a reasonable combination of the starting point polar angles and the angles of the overall metallic Fermat Spirals that constitute the meta-device. Thus, a finite-energy airy beam with a deflection angle of 10° is generated on the interface of the waveguide and the air, as shown in Figure 1b. For the circularly polarized illumination cases, following the spin-decouple nature of the meta-atoms, independent phase gradients for the LCP and RCP incidence components are respectively assigned by altering the aforementioned two degree of freedom (DOF). For the LCP incidence case, the combined phase gradients along the *x* and *y* axis result in the coupling of the PW to the SW with a focused wavefront, while for the RCP incidence case, the corresponding phase gradient forces the PW illumination to reflect back to the upper free space with a designed angle instead of being converted to the SW as shown in Figure 1a, which is dominated by the phase gradient of the meta-coupler along the *x*-axis.

The spin-decoupled metasurface [23,24,25,26,27,28] is one of the current research foci in multi-channel encryption display [29,30,31], multi-functional metaplate [32], and vortex modes detection [33], etc. It is commonly implemented by combining the PB phase with the propagation phase or detour phase. These approaches usually depend on multi-DOFs optimization, including the physical dimensions and orientation angles of the individual meta-atom, or even the relative position between neighboring elements in the array. Although the emergence of deep learning techniques [34,35,36] demonstrates great potential in various fields, including metasurface, even complex-amplitude modulation and demodulation, the multi-DOFs optimization procedure requires a lot of computing resources and is time-consuming for large-scale meta-device applications. On the other hand, chiral structures are exploited to achieve spin-decoupled full-range phase modulation by combing the AA phase with the PB phase [28,37]. This approach works by simply altering the polar angle of the starting and ending point of the chiral structures and achieves a spin-decouple property at the meta-atom level, which makes it much more compact for miniaturization and integration of the meta-devices. Furthermore, as only two angular DOFs are needed, the design processes for large-scale and complicated meta-devices get simplified by avoiding the time-consuming parameter searching process.

**Figure 2:**
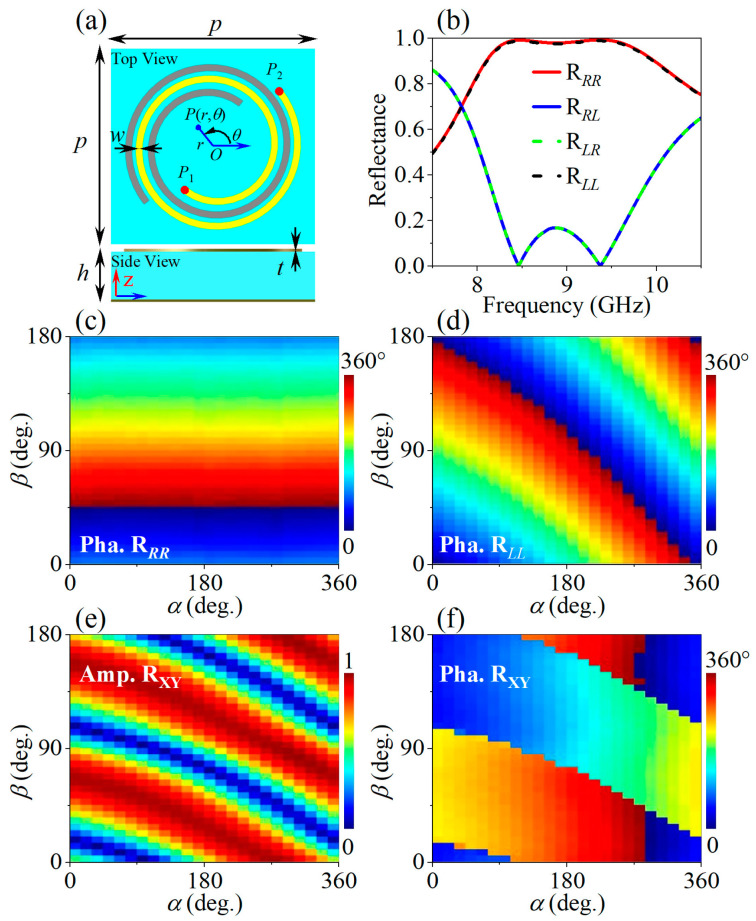
(**a**) Schematic of the meta-atom structure; (**b**) Calculated reflectance spectra of the meta-atom with parameters of (*α*, *β*) = (10°, 0°) under circular polarization basis; Calculated reflection phase of the (**c**) LCP-to-LCP component and (**d**) the RCP-to-RCP component versus the polar angle *α* of the starting point and the overall orientation angle *β*; Calculated (**e**) amplitude and (**f**) phase of the Y-to-X reflection component versus *α* and *β*.

The meta-atom is shown in Figure 2a, which consists of two metallic layers of thickness *t* and a dielectric spacer (F4B, *ε_r_* = 2.65, *tg* = 0.0017) of thickness *h*. The bottom metallic layer is a continuous copper film as the reflective backboard. The upper layer consists of two centrosymmetric Fermat Spirals of line width *w*, saying that the gray one is constructed by rotating the yellow one by 180° around the original point. Here we take the yellow one as an example, the trace of which follows Fermat’s spiral equation

(1) $$r=a\theta^{1/2}.$$

The polar coordinates (*r*, *θ*) are defined as shown in Figure 2a and *a* represents the scaling factor of the radius. The polar angle of the starting point *P*_1_ and ending point *P*_2_ are *θ*_1_ = *α* and *θ*_2_ = 810°, respectively. Besides, an additional in-plane rotation angle *β* around the original point is applied to the overall spiral. First of all, for the structure parameter of an unrotated state, (*α*, *β*) = (10°, 0°), the calculated reflectance under periodical boundary conditions is shown in Figure 2b. The parameters are adopted as the period *p* = 8.5, the thickness of the spacer *h* = 2 mm, the thickness of the copper layer *t* = 0.018 mm, the spiral line width *w* = 0.2 mm, and the scaling factor *a* = 1. The calculation of the unit cells and the following meta-devices in this work are all implemented with the commercial software *Eastwave* (version 7.0) [38]. In the meta-atom calculation, the periodic boundary conditions for the *x* and *y* directions, the PML boundary condition for the +*z* direction, and the PEC boundary condition for the −*z* direction is applied. To ensure the convergence and accuracy of the calculation, the meshgrid density is set up to 20 lines per wavelength. The waveguide port is used to analyze the scattering behaviors of the orthogonal linear or circular polarization states. As we can see, the co-polarization reflectance of greater than 98% in 8.5~9.5 GHz is realized for both LCP and RCP incidence. For the center frequency of about 9 GHz, the co-polarization reflection phases are further analyzed in the parameter space of *α* ∈ [0, 360°] and *β* ∈ [0, 180°), as shown in Figure 2c,d. It is obvious that the co-polarization reflection phase for the RCP incidence case responds only to the overall orientation angle *β*, which follows the PB phase modulation scheme. On the other hand, it does not change with respect to the polar angle *α* of the starting point *P*_1_; while, for the LCP incidence case, the co-polarization reflection phase shows both the PB phase dependence that is related to the overall orientation angle *β*, and the AA phase dependence resulting from the change of the polar angle of the spiral’s starting point. Basically speaking, the full-range co-polarization reflection phase control abilities for both the LCP and RCP incident waves are realized by combing the PB phase and AA phase mechanisms, noting that the accompanying reflectance is larger than 89% in the frequency band of interest.

According to the calculated reflection properties, the reflection Jones calculus of the meta-atom under circular polarization basis in the frequency band of interest can be expressed as

(2) $$M_{}^{circ}=\left(\begin{array}{cc} R_{LL} & R_{LR}\\ R_{RL} & R_{RR} \end{array}\right)=\left(\begin{array}{cc} e^{i\left[f\left(\alpha \right)-2\beta \right]} & 0\\ 0 & e^{i2\beta } \end{array}\right),$$

where *e^if^*^(*α*)^ represents the LCP co-polarization reflection phase factor that is determined by the polar angle of the starting point of the Fermat Spiral, and *e^iσ^*^2*β*^ (*σ* = ±1 for RCP and LCP incidence, respectively) shows the PB phase factor introduced by the overall rotation of the structure. Equation (2) can be rewritten as

(3) $$M_{}^{circ}=e^{i\varphi }\left(\begin{array}{cc} e^{-2i\phi } & 0\\ 0 & e^{2i\phi } \end{array}\right).$$

*φ* = *f*(*α*)/2, *ϕ* = *β − f*(*α*)/4 are two independent variables that are related to *α* and *β*. Through basis transformation, the Jones calculus under linear polarization basis can be derived as

(4) $$\begin{array}{ll} M_{}^{lin} & =\left(\begin{array}{cc} R_{XX} & R_{XY}\\ R_{YX} & R_{YY} \end{array}\right)\\ & =e^{i\varphi }\left(\begin{array}{cc} \cos2\phi & \sin2\phi \\ \sin2\phi & -\cos2\phi \end{array}\right)\\ & =e^{i\varphi }R\left(-\phi \right)\left(\begin{array}{cc} 1 & 0\\ 0 & -1 \end{array}\right)R\left(\phi \right), \end{array}$$

where $R\left(\phi \right)=\left(\begin{array}{cc} \cos\phi & \sin\phi \\ -\sin\phi & \cos\phi \end{array}\right)$ denotes the rotation matrix. The last three factors in Equation (4) describe an idle half-wave plate with its optical axis rotated by *ϕ*, while the first factor shows a global phase factor for all the reflection components. It is clear that, for all the elements in *M^lin^*, the parameter *ϕ* results in full-range amplitude modulation with an accompanying phase of 0 or *π*, while the parameter *φ* brings the independent full-range phase modulation. This is possibly explained by the nature of the physics system that is described by Equation (4); that is, as an ideal half-wave plate rotated by *ϕ*, the optical element totally reflects the incident linear polarized incident wave and changes its polarization direction by 2*ϕ*, hence resulting in the amplitude modulation of the observed projection component in the *x* or *y* direction. Meanwhile, the isotropic propagation phase introduced by *φ* for all the reflection components mimics an offset reference plane from the surface of the equivalent optical element.

Now briefly speaking, multiple amplitude and phase modulation functions emerge based on the proposed metallic spiral structure by properly tuning two DOFs of the starting point polar angle *α* ∈ [0, 360°] and the overall orientation angle *β* ∈ [0, 180°). Firstly, the full-range co-polarization reflection phase control can be independently realized for the LCP and RCP incidence cases. Secondly, for both the co-polarization and cross-polarization reflection components of linear polarization incidence cases, full-range reflection amplitude control covering [0, 1] and full range reflection phase control covering [0, 360°) can be realized independently, which is vital for the complex wavefront tailoring in both PW and SW applications.

## 3. Meta-Device Design

### 3.1. Supercell Analysis

**Figure 3:**
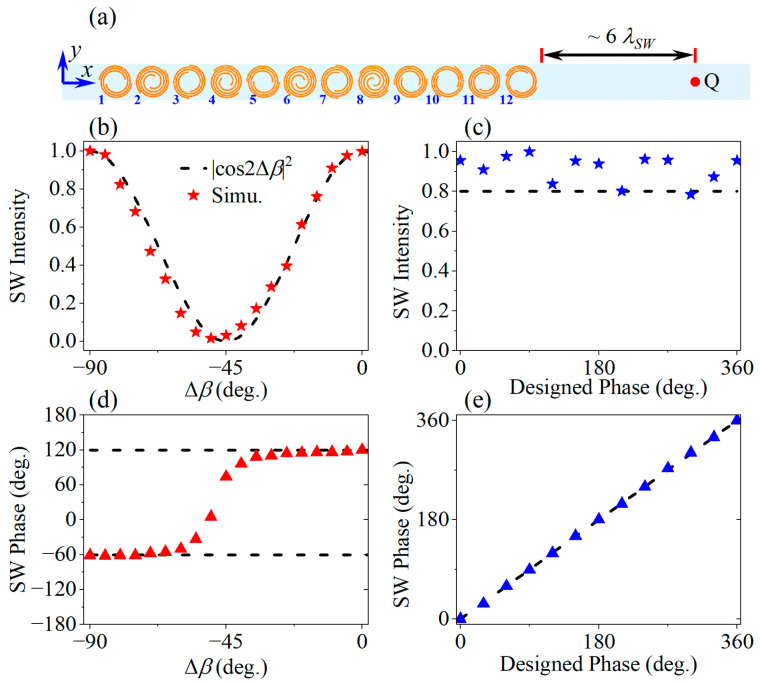
Independent amplitude and phase modulation ability for the cross-polarization component under linear polarization illumination. (**a**) Configuration of the supercell; (**b**) Continuous amplitude modulation and (**d**) accompanying binary phase based on the overall orientation angle. The red triangles in (**d**) represent the calculated phase values; (**e**) Continuous phase modulation and (**c**) the near-stable accompanying amplitude, where the blue pentagrams in (**c**) and the blue triangles in (**e**) represent the calculated intensity and phase values, respectively.

With our meta-atoms’ reflection amplitude and phase control properties fully revealed, we now use them to demonstrate two kinds of metasurface coupler with polarization-dependent SW modulation functionalities. The first kind of metasurface coupler, including two examples with different functions, is designed for advanced SW coupling together with independent amplitude and phase control of the SW wavefront in the microwave regime. The second kind of metasurface coupler is designed for demonstration of the fundamental spin-decouple nature and its bi-functional wavefront tailoring ability. As we know, the surface plasmon polaritons cannot be excited in this regime, so we adopt the guided SWs that are supported in a dielectric waveguide. As shown in Figure 3a, the metallic-backed dielectric waveguide has a thickness of 2 mm in the z direction, the same as the meta-atom spacer. Considering the center frequency 9 GHz of the meta-atoms, the wave vector of SW mode can be calculated following the dielectric waveguide theory, which results to be *k_SW_* = 1.0296*k*_0_ with *k*_0_ being the wave vector in free space. Following the supercell approach [8], we construct the momentum matching by introducing the phase gradient at the supercell level. As aforementioned, by altering the initial phase of the leftmost element of the supercell or uniformly tuning the reflectance, supercells with different complex amplitudes can be constructed. By consecutively arranging a number of such supercells side by side, the specific aperture distribution can be realized. Figure 3a depicts the supercell configuration of this work. It consists of 12 unitcells along the *x*-axis, the reflection phase distribution of which is adopted as *φ_x_* to fulfill the phase gradient of |Δ*φ_x_*/Δ*x*| = *k_SW_* for the SW coupling. Hence, the phase difference needed for adjacent unitcells can be calculated as around 94.6°.

We first calculate and analyze the properties of the supercell. A *y*-polarized plane wave source is set to generate a normal incident wave, which is coupled into the SW modes to the dielectric waveguide by the supercell and then propagate rightwards along the +*x* direction in Figure 3a. For the supercell calculation, the boundary conditions for the *y* and *z* directions are the same as for the meta-atom calculation, while for the *x* directions the PML are adopted. Besides, a planewave port is applied at the simulation boundary of the +*z* direction and an electric field monitor that covers the entire simulation region is added to record the three-dimensional vector electric field distribution information. The SWs are characterized by sampling the *Ez* filed component at 1 mm above the dielectric waveguide and 200 mm (~6 *λ_SW_*) away from the right boundary of the coupler, as denoted by point Q in Figure 3a.

Firstly, for evaluating the amplitude modulation property, a supercell is constructed by assigning the cross-polarization reflection coefficient distribution of *R_XY_* = exp[−*ik_SW_*(*n_x_* − 1)*p*], where *n_x_* = 1, 2, 3, …, 12 denotes the index of the 12 meta-atoms that constitute the supercell, noting that *R_XY_* indicates the perfect cross-polarization conversion by assuming a unit reflection amplitude. Then, the parameters (*α_nx_*, *β_nx_*) of each meta-atom in the supercell can be determined according to the relationship shown in Figure 2e,f. Next, we keep the parameter *α_nx_* constant and synchronously rotate all the meta-atoms in the supercell by an angle of Δ*β* to get a new supercell with parameters (*α_nx_*, *β_nx_* + Δ*β*). After calculating one set of supercells for Δ*β* ∈ {−90°: 5°: 0}, the *E_z_* components at the sampling position Q are extracted. Figure 3b,d shows the SW intensity |*E_z_*|^2^ (normalized to the corresponding value when Δ*β* = 0) and phase arg(*E_z_*), respectively. It is clear that SW intensity varies regularly and shows great agreement with the |cos2Δ*β*|^2^ relationship represented by the dashed line. At the same time, the SW phase remains near constant in the left and right half intervals, respectively, noting that the phase inversion occurs where the intensity vanishes, as is predicted in Equation (4).

On the other hand, for evaluating the amplitude modulation property, we construct another set of supercells with the cross-polarization reflection coefficient of *R_XY_* = exp{−*i*[*k_SW_*(*n_x_* − 1)*p* + *φ_target_*]}, where *φ_target_* ∈ {0: 30°: 360°} denotes the desired phase of the coupled SW wavefront. By conducting the same operation as above-mentioned, we get the calculated normalized intensity and phase of the SW at the sampling position Q, as shown in Figure 3c,e. Notice that the calculated phase values agree very well with the goals and the accompanying normalized amplitudes are basically above 0.8 even if experiencing a certain amount of fluctuation. Thus, the results of the two sets of supercell calculation verify the excellent independent SW amplitude and phase control ability of the proposed meta-atom and supercells.

### 3.2. Meta-Device Design for Airy Beam Generation

**Figure 4:**
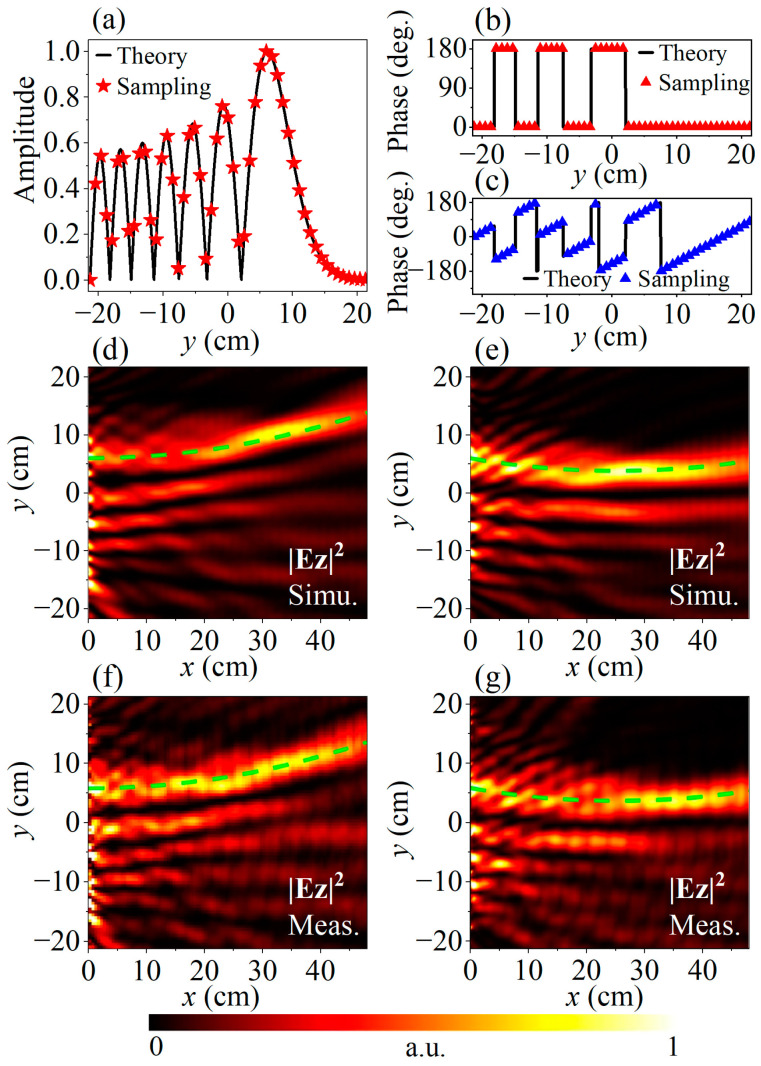
The (**a**) amplitude and (**b**) phase distribution for SW airy beam generation; (**c**) Phase distribution for SW airy beam generation with 10° deflection; The (**d**) simulation and (**f**) experiment results of the airy beam without deflection; The (**e**) simulation and (**g**) experiment results of the airy beam with 10° deflection. The green lines reveal the corresponding main lobe trajectories.

Next, we use the aforementioned supercells to construct two metasurface couplers of the first kind for SW airy beam generation with and without deflection, respectively. As a paraxial self-accelerating and non-diffraction beam, airy beam [39] is promising in imaging [40], particle manipulation [41], and light bullets [42,43]. Without losing generality, we focus on one-dimensional (1-D) SW airy beam deflection for proof of principle, while the proposed meta-atom and supercell are ready for generating two-dimensional SW airy beams. For 1-D finite energy airy beam that is normally lunched and propagates along the *x*-axis in the *xoy* plane, the complex electric field distribution on its initial position can be described as

(5) $$U\left(y,0\right)=A\cdot Ai\left(\frac{\left(y-y_{0}\right)}{w}\right)\exp\left(\frac{\alpha \left(y-y_{0}\right)}{w}\right),$$

where *A* is the amplitude, *Ai* represents the Airy function, *α* is the decay factor, *w* is the aperture scaling factor, and *y*_0_ is a reference coordinate for normalization. For devices that generate airy beam with a normal lunch angle, only continuous amplitude modulation in [0, 1] and two segments of 0 and *π* phase modulation are required [44], as depicted by the solid lines in Figure 4a,b where the parameters are chosen to be *A* = 1, *y*_0_ = 12 mm, *w* = 28.5 mm, *α* = 0.01. Therefore, in order to verify the full-range independent amplitude and phase modulation property of the proposed structure, the second meta-device is constructed with an additional linear phase gradient *k_y_* introduced to deflect the lobes of the airy beam by 10° towards the +*y* direction, which is determined by *k_y_* = *k_SW_*∙sin10°. The phase of the excitation field for the deflected airy beam is shown in Figure 4c, while the amplitude is the same as that for the undeflected one shown in Figure 4a.

Now, we can construct these meta-devices following the aforementioned supercell design process. Each of the meta-devices consist of *N_y_* = 51 supercells along the *y* direction, so the total meta-atoms needed is *N_y_* × *N_x_* = 612. Following Equation (5), the cross-polarization reflection amplitude *R_XY_* of each meta-atom of the device depends on its *y*-coordinate, which is achieved by adding up the 0 or *π* phase and the linear gradient phase −*k_y_n_y_p*, where *n_y_* = −25, −24, …, 0, …, 24, 25. Specifically, the latter term vanishes for the case of norm launch of the airy beam. Besides, the cross-polarization reflection amplitude *R_XY_* of each meta-atom keeps uniform in the *x*-direction, but with a linear gradient phase −*k_SW_*(*n_x_* − 1)*p* for PW-to-SW coupling. Finally, the total phase can be derived by summing the *x* and *y* components. After obtaining the total cross-polarization reflection amplitude and phase of *R_XY_*, the structure parameters (*α_nx,ny_*, *β_nx,ny_*) of each meta-atom can be derived following the aforementioned process.

In the full-wave simulation of the meta-devices, a plane wave source with *y*-polarization is adopted. A near-field monitor is set to record the 3-D vector electromagnetic field over the whole simulation region, from which the *E_z_* component on the horizontal plane 1 mm over the meta-device aperture is extracted. Here the boundary condition setup is the same as the supercell calculation, except that the boundary conditions for the *y* direction is also set as PML. The calculated SW airy beam field intensity with and without deflection at 9 GHz is depicted in Figure 4d,e, respectively, where the green dashed lines reveal their main lobe traces, noting that the calculated and measured intensities in this work are normalized to their corresponding global maximums. As is shown in Figure 4f,g, all results agree well with the calculated ones, each illustrating a noticeable parabolic trajectory with or without deflection angle, respectively, as depicted by the green dashed lines. These results confirm the feasibility of the proposed design method of full-range complex amplitude modulation over the coupled SW. It is worth noting that some stripes exist in the measured *E_z_* fields in Figure 4 and they are supposed to be mainly caused by the inevitable scattering from the edges of the finite-sized samples, which form the standing waves in the measured areas, while these interferences are too weak to destroy the main lobe propagation of the airy beams.

**Figure 5:**
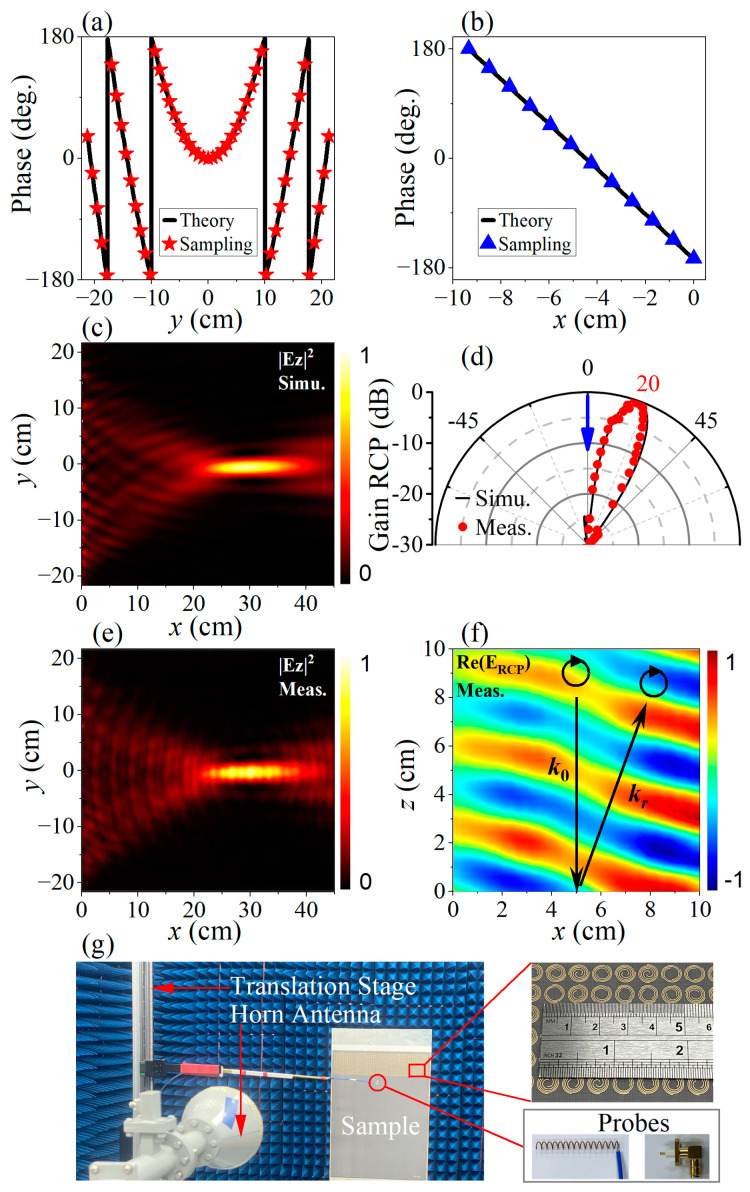
Spin-decoupled bi-functional meta-device design. (**a**) Phase distribution along the *y*-direction for SW focusing; (**c**) Simulated and (**e**) measured SW focusing; (**b**) Phase distribution along the *x*-direction for PW beam deflection; (**d**) Simulated and measured far-field PW beam deflection results, where the blue arrow indicates the wavevector of the incident wave; (**f**) Simulated near-field PW deflection results. The black arrows indicate the wavevector of the incident and deflected PWs, respectively. The black circular arrows represent the polarization states of the incident and deflected PWs, respectively. (**g**) Near field sampling experimental setups. Insets: (**upper**) photo of the spin-decoupled bi-functional meta-device sample and the probes for (**bottom left**) PW near-field and (**bottom right**) SW near-field sampling.

The devices analyzed here and in the following section are manufactured using the traditional printed circuit board (PCB) technique on F4B substrates (Taconic TLT-6). The PCB technique is a mature process and can provide precise control of the pattern shapes and layer alignment. The devices share the same overall dimensions of *L_x_* × *L_y_* = 590 mm × 440 mm. The anechoic environment used for field measurement is depicted in Figure 5g, where the horn antenna is adopted as the source with linear or circular polarization that depends, and the probes are mounted on a translation stage for a near-field scan with Keysight PNA-X 5242A Network Analyzer (Penang, Malaysia). Specifically, a small monopole antenna is adopted for SW measurement, as shown by the bottom right inset of Figure 5g, while a small helix antenna is used for RCP PW sampling, as shown by the bottom left inset of Figure 5g. A global scanning step of 5 mm is adopted for all the near-field sampling experiments in this paper. On the other hand, the far-field measurement is also conducted in an anechoic chamber using the standard process, in which the sample is placed on a sample stage that rotates by step of 1° with a bounded horn antenna as the normal incident source. The fixed receiving horn antenna is placed 5 m away from the sample stage to record the reflected RCP signals from the sample.

### 3.3. Polarization Delinked Wavefront Manipulation

In addition, to confirm and verify the spin-decoupled wavefront tailoring properties of the proposed meta-atoms, we design another meta-device with spin-selective functions of the SW focusing and PW deflection for LCP and RCP planewave illuminations, respectively. The overall dimensions of this meta-device are the same as the above airy beam devices and contain *N_x_* × *N_y_* = 12 × 51 meta-atoms.

Firstly, for the LCP illumination cases, the required complex-amplitude distributions on the SW focusing metalens’ aperture can be expressed as

(6) $$\varphi_{focus}^{LCP}\left(y\right)=k_{sw}(\sqrt{y^{2}+f^{2}}-f),$$

where *y* = *n_y_p* is the physical *y*-coordinate of the meta-atoms. In addition, the PW-to-SW coupling phase along the *x*-axis is *φ^LCP^_couple_* = −*k_SW_*, where *x* = (*n_x_* − 1)*p*. Thus, the overall phase distribution for LCP incidence turns out to be

(7) $$\varphi^{LCP}=\varphi_{focus}^{LCP}+\varphi_{couple}^{LCP}.$$

On the other hand, for the RCP incidence case, only a phase gradient along the *x*-axis is needed to deflect the incident wave by an angle *γ* in the *xoz* plane,

(8) $$\varphi^{RCP}=k_{//}x,$$

where *k*_//_ = *k*_0_ sin*γ*. For *γ* = 20°and *f* = 300 mm, structure parameters of all the meta-atoms can be uniquely determined based on *φ^LCP^* and *φ^RCP^* through the interpolation process. That is, for meta-atom (*n_x_*,*n_y_*), its overall orientation angle *β_nx,ny_* is firstly interpolated in Figure 2c to acquire the specific value of *φ^RCP^_nx,ny_*_,_ and the polar angle of the starting point *α_nx,ny_* of the Fermat Spiral is next determined in Figure 2d to meet the specific value of *φ^LCP^_nx,ny_*. All the meta-atoms in the *N_x_* × *N_y_* array can be uniquely determined following this approach. In Figure 5a, the red stars depict the phases of the leftmost column of the meta-atoms array extracted from Figure 2a,b based on the parameters (*α_nx,ny_*_,_ *β_nx,ny_*), whereas the black solid line illustrates the theoretical phase distribution for SW focusing with the focus length of *f* = 300 mm. Similarly, the blue triangles in Figure 5b represents the sampled phases of the elements along the *x*-direction for PW deflection by 20°, and the black solid line represents the corresponding theoretical values. It can be seen that the phase interpolation results obtained by the above process are in good agreement with the target values.

The SW focusing performance is simulated under LCP planewave illumination on the meta-device aperture. The calculated focal length shown in Figure 5c is located at around *x* = 298 mm, which is quite close to the design value of 300 mm. On the other hand, for the RCP incidence case, the calculated far-field pattern of PW beam deflection is shown by the black solid line in Figure 5d. The main lobe clearly points to about 20°, which is consistent with the design target. This spin-dependent bifunctional meta-device is also manufactured with the PCB technique and experimentally measured in an anechoic chamber. The measured results under RCP and LCP illumination are depicted in Figure 5e and the red points in Figure 5f, respectively. The measured data also agree well with the calculated results. In addition, to fully reveal PW beam deflection performances, we also measure the near-field distribution of the co-polarization reflection component. As depicted in Figure 5f, the reflected wavefront basically propagates along the black arrow indicating the designed deflection direction, thus verifying the performance of the meta-device. It is worth mentioning that the measured reflection near-field demonstrates a slightly irregular wavefront. This is believed to be attributed to the weak scattering from the left edge of the sample, which can also be distinguished in the far-field results in Figure 5d, where a small depression appears at about 10°. However, this undesired character does not evidently affect main lobe propagation, and can be suppressed by expanding the distance between the coupling area and the left edge of the sample.

## 4. Conclusions

To summarize, we have presented a metasurface coupler design method for coupling PWs into SWs with a full range of complex-amplitude wavefront modulation. By combining the AA phase and PB phase through altering the two orientation angles of the Fermat Spiral meta-atom, an arbitrary combination of amplitude and phase can be achieved for the linear polarized illumination case. For verification, two SW airy beam generators respectively for norm launch and 10° beam deflection are fabricated and characterized in a microwave regime. The fundamental spin-selective character is also verified with another spin-decoupled bi-functional device. The geometric-phase-based concept significantly reduced design complexity and also provides a compact approach for flexible SW manipulation in integrated optics, optical sensing, and other related fields ranging from microwave to terahertz and optical regimes.

## Data Availability

Data will be made available on request.
